# Spectroscopic and Theoretical Studies of Some 3-(4′-Substituted phenylsulfanyl)-1-methyl-2-piperidones

**DOI:** 10.3390/molecules18077492

**Published:** 2013-06-27

**Authors:** Paulo R. Olivato, Jean M. M. Santos, Bruna Contieri, Carlos R. Cerqueira, Daniel N. S. Rodrigues, Elisângela Vinhato, Julio Zukerman-Schpector, Maurizio Dal Colle

**Affiliations:** 1 Conformational Analysis and Electronic Interactions Laboratory, Institute of Chemistry, University of São Paulo, USP, CP 26077, 05513-970 São Paulo, SP, Brazil; E-Mails: jean.miguel10@gmail.com (J.M.M.S.); brunacontieri@hotmail.com (B.C.); carloscerq@gmail.com (C.R.C.); dannopper@gmail.com (D.N.S.R.); 2 Institute for Environmental, Chemical and Pharmaceutical Sciences, Federal University of São Paulo, UNIFESP, Diadema 09972-270, SP, Brazil; E-Mail: vinhato@iq.usp.br; 3 Chemistry Department, Federal University of São Carlos, CP 676, 13565-905, São Carlos, SP, Brazil; E-Mail: julio@ufscar.br; 4 Department of Chemistry, University of Ferrara, Ferrara 44100, Italy; E-Mail: mau@unife.it

**Keywords:** conformational analysis, infrared spectroscopy, theoretical calculations, 3-(4′-substituted phenylsulfanyl)-1-methyl-2-piperidones, X-ray crystal structure

## Abstract

The analysis of the IR carbonyl bands of some 3-(4′-substituted phenylsulfanyl)-1-methyl-2-piperidones **1**–**6** bearing substituents: NO_2_ (compound **1)**, Br (compound **2**), Cl (compound **3**), H (compound **4**) Me (compound **5**) and OMe (compound **6**) supported by B3LYP/6-31+G(d,p) and PCM calculations along with NBO analysis (for compound **4**) and X-ray diffraction (for **2**) indicated the existence of two stable conformations, *i.e.*, *axial* (*ax*) and *equatorial* (*eq*), the former corresponding to the most stable and the least polar one in the gas phase calculations. The sum of the energy contributions of the orbital interactions (NBO analysis) and the electrostatic interactions correlate well with the populations and the ν_CO_ frequencies of the *ax* and *eq* conformers found in the gas phase. Unusually, in solution of the non-polar solvents *n*-C_6_H_14_ and CCl_4_, the more intense higher IR carbonyl frequency can be ascribed to the *ax* conformer, while the less intense lower IR doublet component to the *eq* one. The same ν_CO_ frequency trend also holds in polar solvents, that is ν_CO (*eq*)_ < ν_CO (*ax)*_. However, a reversal of the *ax*/*eq* intensity ratio occurs going from non-polar to polar solvents, with the *ax* conformer component that progressively decreases with respect to the *eq* one in CHCl_3_ and CH_2_Cl_2_, and is no longer detectable in the most polar solvent CH_3_CN. The PCM method applied to compound **4** supports these findings. In fact, it predicts the progressive increase of the *eq*/*ax* population ratio as the relative permittivity of the solvent increases. Moreover, it indicates that the computed ν_CO_ frequencies of the *ax* and *eq* conformers do not change in the non–polar solvents *n*-C_6_H_14_ and CCl_4_, while the ν_CO_ frequencies of the *eq* conformer become progressively lower than that of the *ax* one going from CHCl_3_ to CH_2_Cl_2_ and to CH_3_CN, in agreement with the experimental IR values. The analysis of the geometries of the *ax* and *eq* conformers shows that the carbonyl oxygen atom of the *eq* conformer is free for solvation, while the O_[CO]_^…^H_[*o*-Ph]_ hydrogen bond that takes place in the *ax* conformer partially hinders the approach of the solvent molecules to the carbonyl oxygen atom. Therefore, the larger solvation that occurs in the carbonyl oxygen atom of the *eq* conformer is responsible for the observed and calculated decrease of the corresponding frequency. The X-ray single crystal analysis of **2** indicates that this compound adopts the most polar *eq* geometry in the solid. In fact, in order to obtain the largest energy gain, the molecules are arranged in the crystal in a helical fashion due to dipole moment coupling along with C-H^…^O and C-H^…^π_Ph_ hydrogen bonds.

## 1. Introduction

The analysis of the infrared (ν_CO_) band and theoretical B3LYP/6-311++G(d,p) calculations of some *N*,*N*-diethyl-2-[(4′-substituted) phenylsulfanyl acetamides Et_2_NC(O)CH_2_SC_6_H_4_-Y (Y=OMe, Me, H, Cl, Br, NO_2_) [1] indicated the existence of two pairs (*anti* and *syn*) of *cis* (*c*) and *gauche* (*g*) conformers in the gas phase, with the *gauche* conformers being significantly more stable (*ca.* 90%) than the *cis* ones. The *anti*-geometry is more stable than the *syn* one for each pair of *cis* and *gauche* conformers. The summing up of the σ_C-S_→π*_C=O_, π_C=O_→σ*_C-S_, π*_C=O_→σ*_C-S_ (NBO) orbital interactions is the main factor that stabilises the *gauche* conformers to a larger extent than the *cis* ones for which these interactions are absent. The similarity of the carbonyl frequencies in the gas phase for the *anti* and *syn* pairs of the *cis* and *gauche* conformers originates a carbonyl doublet in solution for which the less intense, higher frequency component is ascribed to the pair of the *cis* conformers and the lower frequency component corresponds to the more stable pair of the *gauche* conformers.

In solution of the non-polar solvents (*n*-C_6_H_14_ and CCl_4_), the least polar *gauche* conformers (*ca*. 85%) are predominant with respect to the most polar *cis* ones. In the polar solvent, CHCl_3_, a decrease of the *gauche*/*cis* population ratio is observed, *i.e.*, 60% / 40%, and in the highly polar solvent, CH_3_CN, the most polar *cis* conformer is the only one present. This trend was supported by the PCM solvation model performed by the B3LYP/6-311++G(d,p) method.

Theoretical and spectroscopic (IR, NMR and microwave) studies [2,3,4,5] of 2-piperidones showed that the piperidone ring lies in a half-chair or slightly twisted half-chair conformation. X-ray single crystal structure determination of the 3-chloro-2-piperidone [6] has shown that the piperidone ring is in a slightly twisted half-chair conformation with the 3-substituent assuming the *quasi-equatorial* [Cl-C-C=O (dihedral angle) = −30°] geometry. Moreover, our theoretical and X-ray single crystal studies of some 3-(4′-substituted phenylsulfonyl)-1-methyl-2-piperidones [7,8] have shown that, in these compounds, the 2-piperidone ring assumes a slightly distorted half-chair conformation. In the gas phase the 3-substituent adopts the *quasi-equatorial*, *sin-clinal* and *quasi-axial* geometries with respect to S-C-C=O dihedral angles of *ca*. 31°, 46° and 71°, respectively, while in the solid state the preferred geometry is *quasi-axial* with respect to the S-C-C=O dihedral angle of *ca*. 72°.

**Scheme 1:**
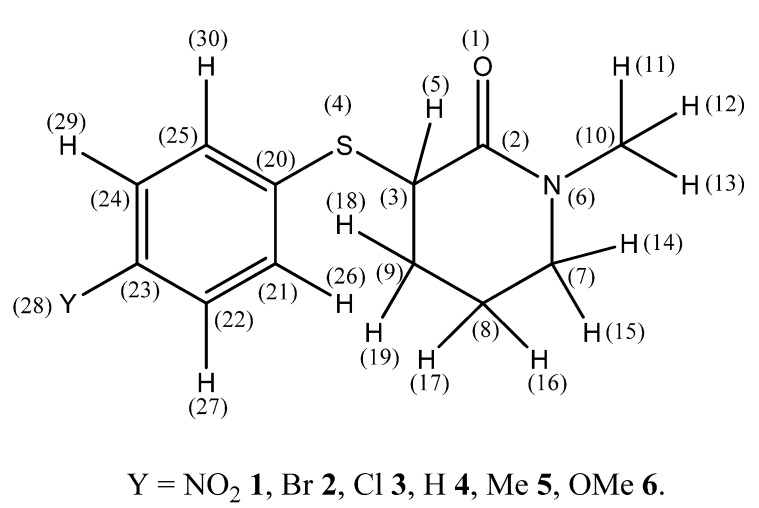
Atom labeling of 3-(4′-substituted phenylsulfanyl)-1-methyl-2-piperidones and definition of relevant torsion angles.

Aiming to throw more light on the nature of the orbital and electrostatic interactions that influence the stability of the *cis*-*gauche* conformers of 2-(4′-substituted)-phenylsulfanylamides [1], this paper reports the IR study of some six-member ring lactams, *i.e.*, 3-(4′-substituted phenylsulfanyl)-1-methyl-2-piperidones bearing in the 4′ position the substituents, *i.e.*, Y = NO_2_ (compound **1**), Br (compound **2**), Cl (compound **3**), H (compound **4**) Me (compound **5**) and OMe (compound **6**) (Scheme 1) along with density functional theory (DFT), polarizable continuum model (PCM) and natural bond orbital (NBO) calculations for **4** taken as the representative compound for the whole series, along with the X-ray diffraction analysis of **2**. These compounds were chosen taking into account some conformational rigidity of the piperidone ring, which allows the 3-substituent to assume almost exclusively the *quasi*-*equatorial* and *axial* conformations. 

α = O(1)-C(2)-C(3)-S(4)

β = C(2)-C(3)-S(4)-C(20)

γ = C(3)-S(4)-C(20)-C(25)

δ = O(1)-C(2)-N(6)-C(10)

θ = O(1)-C(2)-N(6)-C(7)

θ’ = O(1)-C(2)-C(3)-C(9)

ω = C(2)-N(6)-C(7)-C(8)

ω’ = C(2)-C(3)-C(9)-C(8)

## 2. Results and Discussion

**Figure 1:**
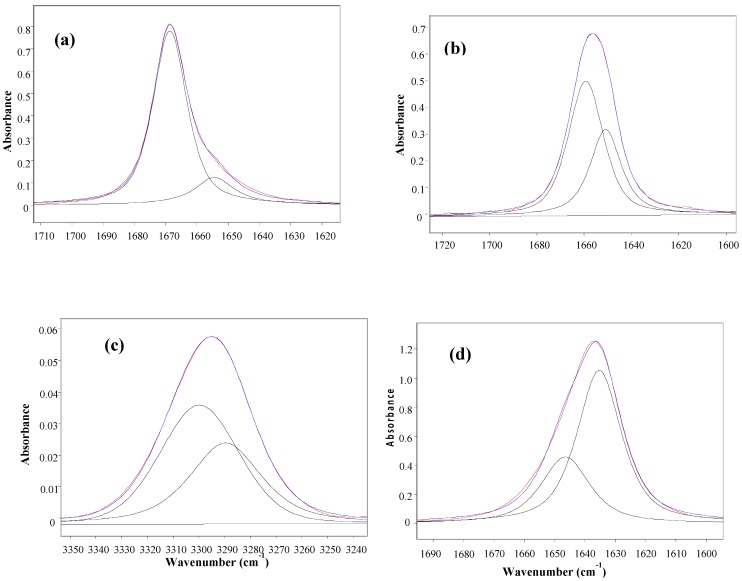

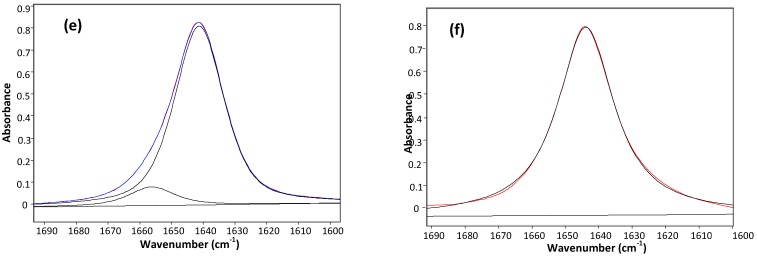
IR spectra of 3-(phenylsulfanyl)-1-methyl-2-piperidone(**4**) showing the analytically resolved carbonyl stretching band in: *n*-hexane (**a**), carbon tetrachloride [fundamental (**b**) and first overtone (**c**)], chloroform (**d**) dichloromethane (**e**) and acetonitrile (**f**).

Table 1 displays the stretching frequencies and the absorbance percentage of the analytically resolved carbonyl band components for compounds **1**–**6** in solvents of increasing relative permittivity [9], *i.e.*, *n-*C_6_H_14_ (ε = 1.9), CCl_4_ (ε = 2.2), CHCl_3_ (ε = 4.8), CH_2_Cl_2_ (ε = 9.1) and CH_3_CN (ε = 38). A doublet is shown in *n*-hexane for derivatives **2**–**6** and in CCl_4_ (fundamental and first overtone regions) for derivatives **1**–**6**, with the higher frequency component being the more intense, *i.e.*, *ca*. 80% and *ca*. 58%, respectively. However, as the relative permittivity increases, a reversal of the carbonyl doublet intensity is observed, with the lower frequency component progressively increasing up to *ca.* 70% in CHCl_3_, *ca*. 88% in CH_2_Cl_2_ and emerges as a singlet in the highest relative permittivity solvent, CH_3_CN. The solvent effect on the carbonyl band components for **4**, chosen as a prototype for the series **1**–**6** is illustrated in Figure 1. 

The unusual observed solvent effect (Table 1) might not be a clear evidence of the existence of conformational isomerism. However, the occurrence of two carbonyl band components in the first overtone region for **1**–**6** in carbon tetrachloride, with comparable intensity ratios and at frequencies twice that of the fundamental minus *ca.* 15 cm^−1^ (that is two times the mechanical anharmonicity [10]), is indicative of the occurrence of two stable conformations for all compounds in this solvent, ruling out the existence of any vibrational effect in the fundamental transition of the ν_CO_ mode [11,12]. Therefore, the IR data strongly suggest the occurrence of two conformers for compounds **1**–**6** in all solvents, with the exception of CH_3_CN, for which only one conformer is present.

**Table 1:** Frequencies (ν, cm^−1^) and intensities of the carbonyl stretching bands in the IR spectra of 3-(4′-substituted-phenyl)sulfanyl-1-methyl-2-piperidones **1**–**6**.

Compound		Y	*n*-C_6_H_14_		CCl_4_				CHCl_3_		CH_2_Cl_2_		CH_3_CN	
			*ν*	*P*^a^	*ν*	*P*^a^	*ν*^b^	*P*	*ν*	*P*	*ν*	*P*	*ν*	P
**1**		NO_2_	-^c^	-	1660	61	3300	60	1648	43	1656	9	-	-
			-	-	1652	39	3289	40	1637	57	1641	91	1645	100
**2**		Br	1668	84	1658	68	3299	63	1645	33	1652	12	-	-
			1656	16	1650	32	3291	37	1635	67	1641	88	1644	100
**3**		Cl	1668	79	1660	45	3306	49	1647	30	1656	13	-	-
			1656	21	1652	55	3292	51	1635	70	1641	87	1644	100
**4**		H	1669	83	1659	62	3300	59	1647	32	1656	9	-	-
			1654	17	1651	38	3290	41	1635	68	1641	91	1644	100
**5**		Me	1668	72	1657	57	3300	57	1645	16	1652	12	-	-
			1659	28	1651	43	3286	43	1634	84	1640	88	1643	100
**6**		OMe	1668	78	1660	44	3301	41	1647	25	1652	15	-	-
			1657	22	1651	56	3288	59	1635	75	1639	85	1643	100

^a^ Relative intensity of each component of the carbonyl doublet, expressed as a percentage of absorbance; ^b^ First overtone; ^c^ Compound slightly soluble in this solvent.

Aiming to estimate the geometry of the minimum energy conformations in the gas phase, B3LYP/6-31+G(d,p) calculations were performed for compound 4, taken as representative of the whole series **1**–**6**. Moreover, in order to estimate the solvent effect on the minimum energy conformations of **6**, the PCM method was applied to fully optimise the referred conformations in the n-C_7_H_16_, CCl_4_, CHCl_3_, CH_2_Cl_2_ and CH_3_CN solvents, and to compute the corresponding vibrational frequencies. The relevant data obtained for **4** are presented in Table 2.

**Figure 2:**
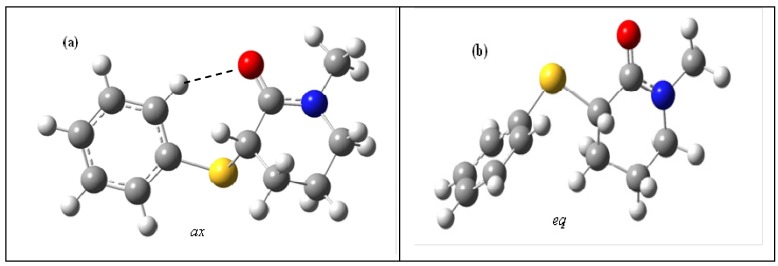
*Axial* (*ax*) (**a**) and *equatorial* (*eq*) (**b**) conformers for **4** obtained at the B3LYP/6-31+G(d,p) level.

Theoretical calculations in the gas phase show the existence of two stable conformations for compound **4**. Due to the significant contribution of the [-O‒C=N+] resonance structure for the hybrid [13,14], the [C(O)NC(10)C(7)] carboxamide moiety (Scheme 1) is almost planar (δ and θ dihedral angles are *ca*. 3° and *ca*. 170°, respectively). This induces the piperidone ring to adopt a slightly distorted half-chair geometry, with the ω and ω′ dihedral angles being *ca*. 27° and *ca*. 51°, respectively (Table 2 and Figure 2) and the 3-phenylsulfanyl substituent assuming the almost *axial* (*ax*) and *equatorial* (*eq*) geometries with respect to the carbonyl group, at α angles of *ca*. 83° and *ca*. 29°, respectively. Moreover, as shown by the θ, θ′, ω and ω′ dihedral angles for the *equatoria*l conformer (Table 2), in the gas phase of **4** and in the solid state of **2**, the substituents at the 4′ position have no influence on the geometry of the lactam moiety.

**Table 2:** Relative energies (E, kJ mol^−1^), dipole moments (μ, D), carbonyl frequencies (ν, cm^−1^), and selected torsion angles (deg) calculated for the minimum energy conformations of 3-phenylthio-1-methyl-2-piperidone **4** in gas phase and in solvents with the PCM method at the B3LYP/6-31+G(d,p) level, and the X-ray geometric data of **2**.

	Conf. ^a^	E ^b^	P ^c^	μ	ν_CO_	Torsion angles/º ^d^							
α						β	γ	δ	θ	θ’	ω	ω'
gas	*ax*	0	96.8	3.62	1713.4	82.0	−101.3	48.2	2.13	167.9	−154.3	23.6	−50.8
	*eq*	8.45	3.2	5.39	1720.1	30.4	−176.5	73.2	−3.90	−170.8	157.1	−23.7	50.8
C_7_H_16_	*ax*	0	84.4	4.08	1696.7	82.0	−101.9	48.4	2.06	168.2	−154.5	23.3	−50.7
	*eq*	4.20	15.6	6.21	1699.0	31.6	−177.4	72.9	−3.74	−172.2	158.2	−22.8	50.3
CCl­_4_	*ax*	0	80.0	4.16	1694.2	81.9	−102.1	49.1	2.06	168.1	−154.5	23.4	−50.7
	*eq*	3.43	20.0	6.37	1694.3	31.1	−176.2	73.3	−3.75	−171.7	157.7	−23.1	50.6
CHCl_3_	*ax*	0.17	48.3	4.61	1676.8	81.9	−102.4	47.7	2.11	168.3	−154.5	23.2	−50.7
	*eq*	0	51.7	7.20	1673.0	33.4	−178.1	72.1	−3.28	−174.9	159.9	−20.6	49.7
CH_2_Cl_2_	*ax*	2.01	30.8	4.89	1668.9	80.6	−104.3	48.5	2.57	169.3	−155.7	23.1	−50.1
	*eq*	0	69.2	7.62	1663.8	32.7	−178.4	71.4	−3.20	−174.3	159.1	−20.8	50.9
CH_3_CN	*ax*	3.93	16.9	5.18	1659.5	80.0	−105.6	48.9	2.60	169.8	−156.2	22.6	−49.8
	*eq*	0	83.1	8.10	1652.3	32.1	−178.4	70.7	−3.11	−173.2	158.5	−21.7	50.2
X-ray ^e^	-	-	-	-	-	31.0 (3)	−176.2 (2)	−155.5(2)	−4.1 (4)	−177.6(3)	157.2(3)	−14.3(4)	53.1 (3)

^a^ Conformer designations *ax* and *eq* refers to the *axial* and *equatorial* conformers, respectively; ^b^ Relative energy; ^c^ Molar fraction in percentage; ^d^ See Scheme 1; ^e^ Refers to compound **2**.

The *ax* conformer is the most stable (*ca*. 97%) and the least polar (*ca*. 3.6 D) and the *eq* conformer is the least stable (*ca*. 3%) and the most polar (*ca*. 5.4 D). Moreover, in the gas phase of 4, the carbonyl frequencies for the *ax* and *eq* conformers are *ca*. 1713 cm^−1^ and *ca*. 1720 cm^−1^, respectively. As expected, this trend is in agreement with the computations of the *N,N*-diethyl-2-[(4′-substituted) phenylsulfanyl acetamides [1] for which, in the gas phase, the less polar gauche conformers are the most stable (lower carbonyl frequency) than the more polar cis conformers (higher carbonyl frequency) (see above). However, the theoretical (gas phase) carbonyl frequencies and the relative population values for the *axial* and *equatorial* conformers for **4** (Table 2) do not match with the IR frequencies and the relative intensities of each carbonyl component in solution (Table 1). Unusually, it seems reasonable to assign the more intense (*ca.* 80%) higher carbonyl frequency component in solution of non-polar solvent (*n*-C_6_H_14_) to the *ax* conformer and the less intense lower carbonyl frequency component to the *eq* one.

Table 2 shows that the torsional angles α-ω’ for the *ax* and *eq* conformers for **4** remain almost the same going from the gas phase to solvents of increasing relative permittivity, *i.e.*, from n-C_7_H_16_ to CH_3_CN. Furthermore, in this direction, there is a progressive decrease of the calculated *ax/eq* conformer population ratio, *i.e*., 97%/3% (gas), 84%/16% (n-C_7_H_16_), 80%/20% (CCl_4_), 48%/52% (CHCl_3_), 31%/69% (CH_2_Cl_2_) and 17%/83% (CH_3_CN) followed by a simultaneous decrease of carbonyl frequency shifts (Δν = ν*_eq_* – ν*_ax_*) between the *eq* and *ax* conformer frequencies that is: *ca*. 7 cm^−1^ (gas), *ca*. 2 cm^−1^ (n-C_7_H_16_), *ca*. 0 cm^−1^ (CCl_4_), *ca*. −4 cm^−1^ (CHCl_3_), *ca*. −5 cm^−1^ (CH_2_Cl_2_) and *ca*. −7 cm^−1^ (CH_3_CN). These trends cannot be justified on the grounds of the dipole moment (μ) analysis as the dipole moment for the *ax* and *eq* conformers increases almost to the same extent going from gas to acetonitrile solution, *i.e*., *ca*. 2.7 D.

A close match was not found between the computed *ax/eq* population ratios (PCM) and the experimental IR ones. However, the progressive decrease of the prevalence of the population of the *ax* conformer over the *eq* one going from *n*-C_7_H_16_ to CCl_4_, along with the progressive increase of the prevalence of the population of the *eq* conformer over the *ax* one going from CHCl_3_ to CH_2_Cl_2_ and to CH_3_CN, seems to be in line with the experimental IR trend of the *ax/eq* population ratio. The fact that the calculated (PCM) carbonyl frequencies for the *ax* and *eq* conformers becomes practically coincident in the non-polar solvents *n*-C_7_H_16_ and CCl_4_, and that the ν_CO_ frequency of the *eq* conformer becomes progressively smaller than the ν_CO_ frequency of the *ax* conformer going from CHCl_3_ to CH_2_Cl_2_ and to CH_3_CN, gives support to the experimental (IR) *ax-eq* carbonyl frequencies assignments (see above).

It should be pointed out that MP2/6-31+G(d,p) calculations for **4** [15] have shown that the population ratio for the *ax/eq* conformers (*ca*. 78%/22%) as well as the torsional angles α-ω’ values for both conformers, are close to those obtained at the B3LYP/6-31+G(d,p) level (Table 2). The computed MP2 carbonyl frequencies indicate that the *ax* and *eq* conformers have almost the same carbonyl frequency of *ca*. 1716 cm^−1^. Moreover the single-point PCM calculations at the MP2 level [16] for the *ax* and *eq* conformers of **4** have shown that the solvent effect on the *ax/eq* population ratio is quite similar to that obtained with the B3LYP method.

Table 3 presents the ChELPG atomic charges [17] for selected atoms computed at the B3LYP/6-31+G(d,p) level for compound **4**, while Table 4 displays the interatomic distances between some selected atoms and the difference between these contacts and the sum of the van der Waals (ΣvdW) radii.

**Table 3:** ChElPG charge (e) at selected atoms obtained at the B3LYP/6-31+G(d,p) level for 3-phenylsulfanyl-1-methyl-piperidone **4**.

Conf.	O(1)	C(2)	S(4)	N(6)	H(26)	H(30)	H(5)	H(11)	H(16)	H(17)
*ax*	−0.557	0.525	−0.321	−0.187	0.131	0.102	0.048	0.088	0.028	0.065
*eq*	−0.537	0.605	−0.320	−0.311	0.103	0.061	0.027	0.058	0.030	0.045

**Table 4:** Selected interatomic distances (Å) for the *ax* and *eq* conformers of 3-phenylsulfanyl-1-methyl-2-piperidone **4** at the B3LYP/6-31+G(d,p) level.

Conf. ^a^	O[1]^...^S[4] ^b^	Δl ^c^	O[1]^...^H[11] ^d^	Δl	O[1]^...^H[26] ^d^	Δl	O[1]^...^H[5] ^d^	Δl
*ax*	3.37	+0.05	2.28	−0.44	2.35	−0.37	2.52	−0.20
*eq*	2.89	−0.43	2.27	−0.45	4.75	+2.03	2.83	+0.11

^a^ Conformer designation; ^b^ Sum of the van der Waals radii = 3.32 Å; ^c^ Difference between non bonded atoms distance and the sum of their van der Waals radii; ^d^ Sum of the van der Waals radii = 2.72 Å.

Due to the decrease of the α dihedral angle going from the *ax* (*ca*. 80°) to the *eq* (*ca*. 31°) conformer there is in the *eq* conformer a short contact between the negatively charged O[1] (≅ −0.55e)…S[4] (≅ −0.32e) atoms whose interatomic distance is significantly shorter than the sum of the van der Waals radii (ΣvdW) (Δl ≅ −0.43Å) while for the *ax* conformer the same contact is close to the ΣvdW radii (Δl ≅ +0.05Å). The referred shorter O^…^S contact of the *eq* conformer should be responsible for the stronger Repulsive Field Effect [11,18] between the C^δ+^=O^δ-^ and C^δ+^─S^δ−^ dipoles in the *eq* conformer, being responsible for both the destabilisation of this conformer relative to the *ax* one and for the higher carbonyl frequency observed in the gas phase of the *eq* conformer with respect to that of the *ax* one whose carbonyl frequency shift (ν*_eq_* – ν*_ax_*) is *ca*. +7 cm^−1^ (Table 2).

As expected from the geometry of the carboxamide moiety of the 1*-*methyl-2-piperidones, there is a short contact between the oppositely charged O[1] (≅ −0.55e)^…^H[11] (≅ 0.09e) atoms whose interatomic distance is significantly shorter than the ΣvdW radii (Δl ≅ −0.44Å; C-H^…^O angle *ca.* 103°) for both*ax* and *eq* conformers (Figure 2). The O[1] (≅ −0.55e) and H[5] (≅ 0.05e) contact is shorter than ΣvdW radii by (Δl ≅ −0.20Å; C-H^…^O angle *ca.* 84°) for the*ax* conformer only. Moreover, as a consequence of the smaller γ dihedral angle in the *ax* conformer (*ca.* 48°) as compared with that of the *eq* one (*ca.* 73°) for **4** (Table 2) the positively charged *o*-phenylsulfanyl hydrogen atom H[26] (≅ 0.13e) gets closer to the negatively charged carbonyl oxygen atom O[1] (≅ −0.55e) Therefore, the interatomic distance becomes shorter than the ΣvdW radii by (Δl ≅ −0.37 Å; C-H^…^O angle *ca.* 148°) for the *ax* conformer and larger than the ΣvdW radii (Δl ≅ +2.0 Å) for the *eq* one.

From these H^δ+…^O^δ-^ contacts analysis it may be concluded that the O[1]^…^H[11] short contact stabilises electrostatically both the *eq* and *ax* conformers to the same extent. However, the O[1]^…^H[5] contact and mainly the O[1]^...^H[26] one stabilise electrostatically the *ax* conformer only.

In order to rationalise the orbital interactions that stabilise the *ax* and *eq* conformers, NBO analysis [19] was performed and the results obtained for selected NBO energy interactions between donor and acceptor orbitals [20] are reported in Table 5.

**Table 5:** Comparison of significant NBO energies (kcal mol^−1^) of the corresponding interacting orbitals for the conformers *ax* and *eq* of 3-phenylsulfanyl-1-methyl-2-piperidone **4** at the B3LYP/6-31+G(d,p) level.

Orbitals	*ax*	*eq*
LP_N6_→π*_C2=O1_	65.3	50.6
LP_O1_→σ*_C2-N6_	25.4	25.5
LP_O1_→σ*_C2-C3_	18.9	20.3
LP_O1_→σ*_C21-H26_	3.0	-
LP_O1_→σ*_C10-H11_	1.0	1.2
LP_O1_→ σ*_S4-C20_	- ^a^	0.9
LP_S4_→ π*_C2=O1_	2.0	-
LP_S4_→ π *_C20-C21_	8.3	2.7
σ_C3-S4_→π*_C2=O1_	4.2	0.7
σ_C3-S4_→σ*_C2-N6_	-	3.4
π_C2=O1_→σ*_C3-S4_	1.7	0.6
π*_C2=O1_→σ*_C3-S4_	5.5	0.8
∑ E^b^	135.3	106.7

^a^ Interaction energy smaller than 0.5 kcal mol^−1^; ^b^ Summing up of the energies of the interacting orbitals.

The most important orbital interaction, the LP_N6_→π*_C2=O1_ corresponding to the [O=C–N ↔ ^−^O–C=N^+^] conjugation, has a value of *ca.* 65 kcal moL^−1^ for the *ax* conformer, significantly higher than that of *ca.* 51 kcal mol^−1^ for the*eq* one. The smaller orbital interaction value for the latter conformer is in agreement with the smaller contribution of the [^−^O–C=N^+^] canonical form to the resonance hybrid due to the strong Repulsive Field Effect between the C^δ+^=O^δ-^ and C^δ+^-S^δ−^ dipoles, as outlined above. On the other hand, the very short O^δ−^[1]^…^H ^δ+^[13] contact with a suitable C─H^…^O angle of *ca*. 148° in the *ax* conformer determines the LP_O1_→σ*_C21-H26_ orbital interaction (*ca.* 3 kcal mol^−1^) (hydrogen bond) in the *ax* conformer. This interaction increases the weight of the polar [^-^O‒C=N^+^] canonical form for the resonance hybrid and is partially responsible for the higher value of the LP_N6_→π*_C2=O1_ orbital interaction cited above. In addition, the higher occupancy value [20] of the σ*_C21-H26_ orbital in the *ax* conformer (0.134 *vs*. 0.014) gives further support for the occurrence of the significant LP_O1_→σ*_C21-H26_ hydrogen bond interaction. 

In the carboxamide moiety, there are two additional higher energy interactions (through bond coupling [21]), LP_O1_→σ*_C2-N6_ and LP_O1_→ σ*_C2-C3_, at mean values of *ca.*26 kcal mol^−1^ and *ca.*20 kcal mol^−1^, respectively, for the *eq* and *ax* conformers.

The more favourable γ dihedral angle of the phenylsulfanyl group (*ca.* 48°) for the *ax* conformer allows a reasonable 3p_S_-π_Ph_* orbital overlap that contributes to a larger stabilisation of the *ax* conformer with respect to the *eq* one [LP_S4_→π*_C20-C21_ orbital interaction of *ca.* 8.3 kcal mol^−1^ (*ax*) and *ca.*2.7 kcal mol^−1^ (*eq*)].

The O[1]^…^H[11] short contacts are responsible for the weak LP_O1_→σ*_C10-H11_ orbital interactions of *ca.* 1.1 kcal mol^−1^ that stabilise to the same extent both conformers. 

It should be pointed out that the *eq* conformer is stabilised through two weak LP_O1_→σ*_S4-C20_ and σ_C3-S4_→σ*_C2-N6_ orbital interactions whose delocalisation energy values are *ca.* 0.9 kcal mol^−1^ and 3.4 kcal mol^−1^, respectively.

The suitable geometry of the *ax* conformer allows the occurrence of superjacent LP_S4_→ π*_C2-O1_ orbital interaction [22] whose value is 2.0 kcal mol^−1^, which is absent for the *eq* conformer. Moreover, in the [O=C-C-S] moiety there are three π(π*)/σ(σ*) orbital interactions: π_C2=O1_→σ*_C3S4_, σ_C3-S4_→π*_C2=O1_ and π*_C2=O1_→σ*_C3-S4_ whose energy values progressively decrease as the π/σ overlap decreases, *i.e.*, they present the maximum value for the *ax* conformer and the minimum one for the *eq* one. In fact, the sum of the energies of these interactions displays the value *ca.*11.4 kcal mol^−1^ and *ca*. 2.1 kcal mol^−1^ for the *ax* and *eq* conformers, respectively. 

In conclusion, since the total sum of the delocalisation energies of the selected interactions has the maximum value of *ca*. 135 kcal mol^−1^ for the *ax* conformer and significantly decreases to *ca*. 107 kcal mol^−1^ for the *eq* one, the NBO analysis and the trend of the electrostatic interactions are in agreement with the higher relative abundance of the *ax* (*ca.* 97%) conformer with respect to the *eq* one, found in the gas phase, whose computed carbonyl frequencies are *ca.* 1713 cm^−1^ and 1720 cm^−1^, respectively.

However, there is no matching between the IR experimental and the theoretical gas phase data. As pointed out above, since a reversal of the IR carbonyl frequencies has been observed experimentally in solution, the higher frequency component has been ascribed to the *ax* conformer, while the lower frequency component to the *eq* one. This behaviour may be rationalised as follows.

**Scheme 2:**
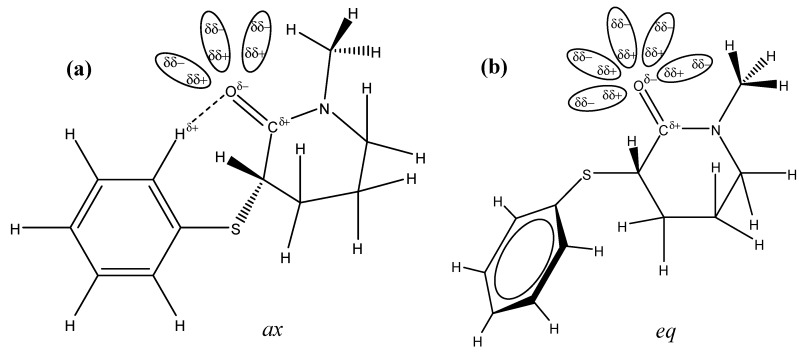
Differential solvent effect for **4** showing that the carbonyl oxygen atom in the *axia*l conformer (**a**) is partially hindered for the approach of the solvent molecules due to [H_(o-Ph)_…O_(CO)_] hydrogen bond in comparison to the *equatoria*l conformer (**b**) for which the carbonyl oxygen atom is free for salvation.

A close inspection of the *ax* (a) and *eq* (b) conformers in the gas phase (Figure 2 and Scheme 2) shows that the *eq* conformer carbonyl oxygen atom is free for solvation, while the hydrogen bond that takes place between the *ortho*-phenylsulfanyl hydrogen atom and the carbonyl oxygen atom lone pair in the *ax* conformer partially prevents the solvent molecules' approach to the carbonyl oxygen atom. 

Therefore, the larger solvation at the carbonyl oxygen atom in the more polar *eq* conformer contributes to the decrease of the carbonyl bond order and, as a consequence, lowers its frequency to a larger extent than that of the *ax* conformer.

**Figure 3:**
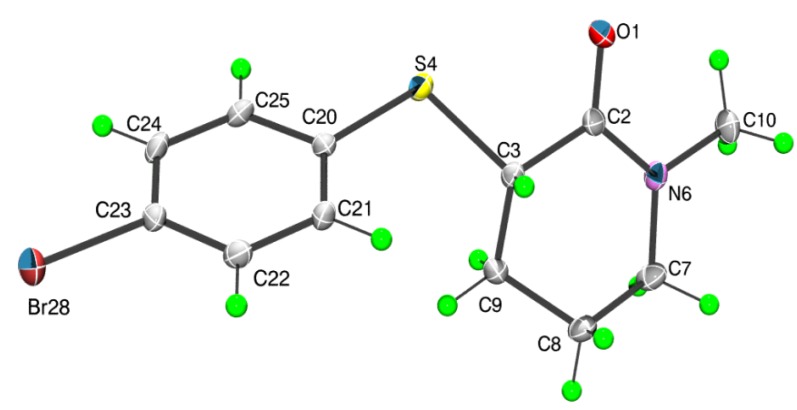
The molecular structure of **2** showing atom labelling scheme and displacement ellipsoids at the 50% probability level (arbitrary spheres for the H-atoms).

X-ray single crystal analysis of **2** showed that in the solid state this compound assumes the *equatorial* geometry with respect to the torsion angle α (Scheme 1) and the 2-piperidone ring, similar to the gas phase of **4**, displays a slightly distorted half-chair conformation (Table 2 and Figure 3).

**Figure 4:**
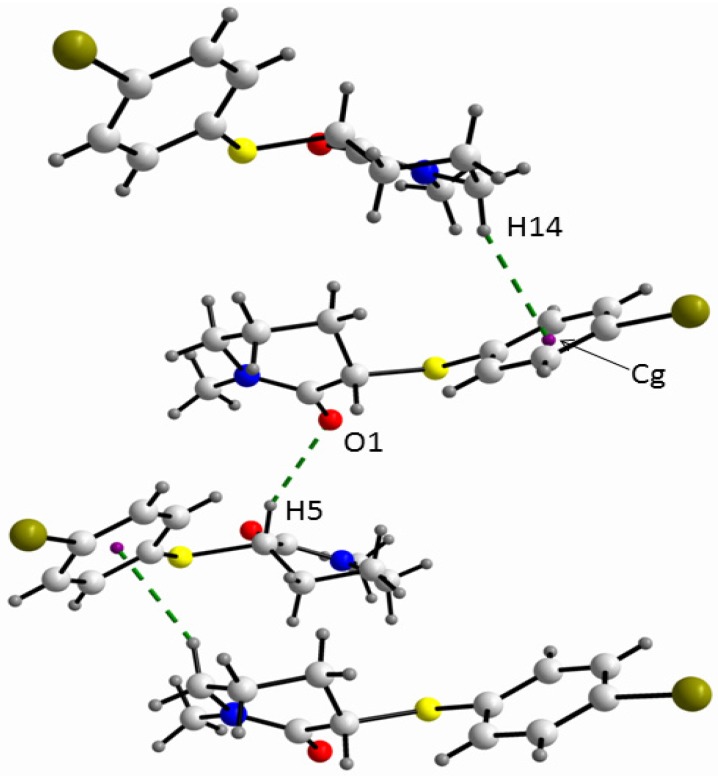
A view of the supramolecular array of **2** where the C-H…O and C-H….π_Ph_ interactions are shown.

The absolute values of the relevant torsional angles (α-ω’) in the solid state of **2** (Table 2) are similar to the corresponding torsional angles in the gas phase of the *equatorial* conformer of **4**, except for the absolute value of the γ angle of *ca.* 155° for **2**, which differs from the γ angle of *ca.* 73° for **4**. The larger γ torsional angle for **2** allows the hydrogen bonding between the π phenyl orbital and the methylene [H14] atom (Figure 4).

In fact, in order to obtain the largest energy gain from the crystal packing, the molecules of the *equatorial* conformer of **2** are stabilised in the crystal through dipole moment coupling along with two hydrogen bond interactions in a helical fashion, *i.e.*, C-H^…^O (C3^i^-H5^i…^O1: C3^i…^O1 = 3.346(3), H5^i…^O1 = 2.43 Å; C3^i^-H5^i…^O1 = 155°; symmetry operation i = 1/2 + x, 1/2-y, 1-z) and C-H^…^π (C7^…^Cg^ii^ = 3.618(4), H14^…^Cg^ii^ = 2.72 Å; C7-H14^…^Cg^ii^ = 155°; symmetry operation ii = x − 1/2, −1/2−y, 1 − z). Additionally, similar to the gas phase of **4**, in the crystal of **2** an intramolecular short contact [C10-H11^...^O1: C^...^O = 2.676(4); H^…^O = 2.23 Å; C-H^...^O = 107°] was found.

## 3. Experimental

### 3.1. Materials

All solvents for IR measurements were spectrograde and used without further purification. The 3-[4′-substituted phenylsulfanyl)]-1-methyl-2-piperidones **1**–**3**, **5** and **6** are new compounds. Derivatives **2**–**6** were obtained from the reaction of 1-methyl-2-piperidone (*a*) with lithium diisopropylamide (*b*), in tetrahydrofuran (THF), followed by the addition of the THF solution of 4-substituted diphenyl disulphide (*c*), in the presence of hexamethylphosphoramide (*d*), in the molar ratio of 1(*a*):2(*b*):1(*c*):1(*d*) as described in the literature for **4** [23]. The appropriate diphenyl disulphide was prepared by the reaction of 4-substituted-thiophenol with bromine in dichloromethane on hydrated silica gel support [24]. The pure phenylsulfanyl piperidones **2**–**6** were obtained in 20–45% yield. The 4′-[nitrophenylsulfanyl]-1-methyl-2-piperidone **1** was obtained as follows: initially, the THF solution of the lithium enolate of 1-methyl-2-piperidone obtained as described above [23] reacted with the tosylate chloride [25] in a 1:1 ratio to obtain the 3-chloro-1-methyl-2-piperidone. This crude product was allowed to react with an ethanolic solution of 4-nitrothiophenol in the presence of equimolar proportion of potassium hydroxide. After usual workup and recrystallization from ethanol-water, the pure compound **1** was obtained in 24% yield. Suitable crystals for X-ray analysis for **2** were obtained by vapour diffusion from chloroform/n-hexane at 283K. The ^1^H- and ^13^C-NMR data for compounds **1**–**6** are collated in Table 6 and elemental analysis results are shown in Table 7.

### 3.2. IR Measurements

The IR spectra were obtained on a FTIR Michelson-Bomem-MB100 spectrophotometer, with 1.0 cm^-1^ resolution. The carbonyl region (1800–1600 cm^−1^) was recorded for *n*-hexane, carbon tetrachloride, chloroform, dichloromethane and acetonitrile solutions, at a concentration of 2.0 × 10^−2^ mol dm^−3^, using a 0.519 mm sodium chloride cell. The carbonyl first overtone region (3500–3100 cm^−1^) was recorded for carbon tetrachloride and chloroform solutions (2.0 × 10^−2^ mol dm^−3^) using a 1.00 cm quartz cell. The overlapped carbonyl bands (fundamental and first overtone) were deconvoluted by means of the Grams/32 curve-fitting program, version 4.04 Level II [26]. The populations of the *axial* and *equatorial* conformers were estimated from the maximum of each component of the resolved carbonyl doublet expressed in percentage of absorbance, assuming equal molar absorptivity coefficients for all the studied compounds 1–6, in each solvent.

**Table 6:** Physical, ^1^H- and ^13^C-NMR data for 3-[(4′-substituted-phenyl)sulfanyl]-1-methyl-2-piperidones 1–6.

Compd.	Y	Mp (°C)	^1^H- and ^13^C-NMR ^a^
**1**	NO_2_	77–78	**^1^H-NMR**: 8.14–8.12 (m, 2H), 7.64–7.63 (m, 2H), 4.06 (t, 1H, *J* = 5.5 Hz), 3.41–3.32 (m, 2H), 2.99 (s, 3H), 2.30–2.27 (m, 1H), 2.09–2.03 (m, 2H), 1.92–1.88 (m, 1H). **^13^C-NMR**: 167.83, 146.07, 145.77, 128.67, 123.86, 49.82, 46.90, 35.36, 28.68, 20.76.
**2**	Br	64–64		**^1^H-NMR**: 7.44–7.40 (m, 4H), 3.82 (t, 1H, *J* = 5.7Hz), 3.33–3.26 (m. 2H), 2.96 (s, 3H), 2.11–2.04 (m, 2H), 1.99–1.94 (m, 1H), 1.82–1.77 (m, 1H). **^13^C-NMR**: 168.36, 134.27, 134.07, 132.19, 121.82, 50.06, 49.02, 35.47, 28.83, 20.59.
**3**	Cl	^b^	**^1^H-NMR**: 7.53–7.51 (m, 2H), 7.30–7.29 (m, 2H), 3.84 (t, 1H, *J* = 5.7Hz), 3.36–3.28 (m, 2H), 2.99 (s, 3H), 2.17–2.08 (m, 2H), 2.02–1.96 (m, 1H), 1.83–1.80 (m, 1H) **^13^C-NMR**: 168.19, 133.77, 133.64, 133.33, 129.06, 49.86, 48.96, 35.27, 28.62, 20.38.	
**4**	H	38–39		**^1^H-NMR**: 7.56–7.54 (m, 2H), 7.32–7.28 (m, 2H), 7.26–7.24 (m, 1H), 3.88 (t, 1H, *J* = 5.0 Hz), 3.30–3.24 (m, 2H), 2.97 (s, 3H), 2.12–2.07 (m, 2H), 1.98–1.94 (m, 1H), 1.77–1.74 (m, 1H). **^13^C-NMR**: 168.47, 134.96, 132.61, 129.12, 127.61, 50.08, 49.03, 35.44, 28.75, 20.46.
**5**	CH_3_	44–45	**^1^H-NMR**: 7.47–7.46 (m, 2H), 7.15–7.13 (m, 2H), 3.83 (t, 1H, *J* = 5,5Hz), 3.34–3.25 (m, 2H), 2.98 (s, 3H), 2.35 (s, 3H), 2.13–2.05 (m, 2H), 1.96-1.92 (m, 1H), 1.80–1.75 (m, 1H) **^13^C-NMR**: 168.37, 137.77, 133.25, 130.79, 129.71, 49.89, 49.21, 35.23, 28.35, 21.12, 20.12.	
**6**	OCH_3_	^b^		**^1^H-NMR**: δ (ppm): 7.51–7.49 (m, 2H), 6.85–6.83 (m, 2H), 3.79 (s, 3H), 3,71 (t, 1H, J = 6,0Hz), 3.25–3,23 (m, 2H), 2.94 (s, 3H), 2.10–2,01 (m, 2H), 1.94–1,89 (m, 1H), 1.75–1,71 (m, 1H). **^13^C-NMR**: 168.36, 159.85, 135.93, 124.61, 114.50, 55.31, 49.89, 49.80, 35.20, 28.28, 20.12

^a^^1^H- and ^13^C- chemical shifts in ppm relative to TMS and coupling constants in Hz at 500 MHz. in CDCl_3_; ^b^ liquid at room temperature; ^c^ From [4]: b.p 130–133° / 0.05 Torr.

**Table 7:** Elemental analysis data for 3-[(4′-substituted-phenyl)sulfanyl]-1-methyl-2-piperidones **1**–**6**.

Compd	Y	Molecular formula		Analysis (%)		
				C	H	N
**1**	NO_2_	C_12_H_14_N_2_O_3_S	Calc.Found	54.1254.34	5.305.23	10.5210.46
**2**	Br						C_12_H_14_BrNOS	Calc. Found	48.0147.91	4.704.77	4.674.79
**3**	Cl	C_12_H_14_ClNOS	Calc. Found	56.3556.26	5.525.22	5.485.27					
**4**	H						C_12_H_15_NOS	Calc. Found	65.1265.12	6.836.56	6.336.46
**5**	CH_3_	C_13_H_17_NOS	Calc. Found	66.3466.23	7.287.20	5.956.01					
**6**	OCH_3_						C_13_H_17_NO_2_S	Calc. Found	62.1262.13	6.826.81	5.575.55

### 3.3. X-ray Measurements

Crystal data for C_12_H_14_BrNOS (**2**): *M* = 300.21, *T* = 290(2) K, orthorhombic, P2_1_2_1_2_1_, *a* = 7.3328(5), *b* = 7.7903(6), *c* = 21.502(2)Å, *V* = 1228.30(17) Å^3^, *Z* = 4, *D*_x_ = 1.623 g cm^−3^, *F*(000) = 608, λ (Mo Kα) = 0.71073, μ = 3.495 mm^−1^, R = 0.0275. 

#### 3.3.1. Data Collection and Processing

Data were collected on a Bruker APEX-II CCD diffractometer using Mo Kα radiation so that θ_max_ = 25.4°, no. of unique data = 2229, no. of parameters = 146, R (1964 data with I ≥ 2σ(I)) = 0.0275, *wR* (all data) = 0.0663. The structure was solved by direct methods [27] and refined with anisotropic displacement parameters for non- hydrogen atoms. The H atoms were geometrically placed (C—H = 0.93–0.98 Å) and refined as riding with *Uiso*(H) = 1.2–1.5*Ueq*(C). The weighting scheme used was *w* = 1/[σ^2^(*F*_o_^2^) + 0.0193*P*^2^ + 0.000*P*] where *P* = (*F*_o_^2^ + 2*F*_c_^2^)/3) with SHELXL-97 [28] on *F*^2^. The programs WinGX [29], ORTEP3 for Windows [30], PLATON [31], MarvinSketch 5.1.10 [32] and DIAMOND [33] were used for geometric calculations and to prepare crystallographic material for publication and depositing. Crystallographic data for the structural analysis have been deposited with the Cambridge Crystallographic Data Centre as CCDC 934425. Copies of this information may be obtained free of charge on application to CCDC, 12 Union Road, Cambridge CB2 1EZ, UK (fax: 44 1223 336 033; e-mail: deposit@ccdc.cam.ac.uk or www: http://www.ccdc.cam.ac.uk). 

### 3.4. Theoretical Calculations

All calculations were carried out (at 298 K) using methods and basis sets implemented in the GAUSSIAN package of programs (G03.E01) [34]. Full geometry optimisations (GJF files for the *eq* and *ax* conformers of 4 are available in the Supplementary material as eq.gjf and ax.gjf) and analytical vibrational frequency calculations were performed on all the orientations with respect to the carbonyl group resultant from a systematic conformational search using the *ab initio* Hartree–Fock method with the 3–21G basis set [35,36,37,38,39], allowing complete relaxation of all internal parameters. The obtained conformations were refined using B3LYP and MP2 methods with the 6-31+G(d,p) basis set [35,36,37,38,39]. Frequency analyses were carried out to verify the nature of the minimum state of all the stationary points obtained. To estimate the solvation effects on the relative stability of the most relevant conformers, a full optimisation and frequency calculations were done starting from the gas-phase optimised structures using the PCM model [40] at the B3LYP/6-31+G(d,p) level as implemented in the GAUSSIAN 03. Additionally, the solvent effect was estimated by PCM single-point calculations MP2/6-31+G(d,p) [41] using the corresponding gas-phase optimised structures. The NBO 3.1 program [19] was used as implemented in the GAUSSIAN 03 package, and the reported NBO delocalisation energies (E2) are those given by second-order perturbation theory. The partial atomic charges were calculated using a grid based method (ChElPG) [42].

## 4. Conclusions

The preferred conformations of some 3-(4′-substituted phenylsulfanyl)-1-methyl-2-piperidones **1**–**6** bearing as substituents NO_2_ (compound **1**), Br (compound **2**), Cl (compound **3**), H (compound **4**) Me (compound **5**) and OMe (compound **6**) were determined by ν_CO_ IR analysis, B3LYP/6-31+G(d,p) and PCM calculations along with NBO analysis (for **4**) and X-ray diffraction (for **2**). Theoretical data indicated the existence of two stable conformations. The 2-piperidone ring of the title compounds assumes a slightly distorted half-chair geometry, while the phenylsulfanyl substituent adopts the almost *axia*l (*ax*) and *equatorial* (*eq*) geometries with respect to the carbonyl group. The *ax* conformer is the most stable (*ca.* 97%), the least polar, and exhibits a lower ν_CO_ frequency. 

Unusually, the higher ν_CO_ frequency component can be ascribed to the *ax* conformer in solution of non-polar (C_6_H_14_ and CCl_4_) and polar solvents (CHCl_3_ and CH_2_Cl_2_). As far as the relative abundance is concerned, the *ax* conformer component progressively decreases with respect to the *eq* one as the relative permittivity increases, and is no longer detectable in the most polar solvent CH_3_CN.

The PCM method was applied to derivative **4** to fully optimise the *ax* and *eq* conformations in all solvents. The torsional angles α-ω’ of the *ax* and *eq* conformers remain practically the same going from the gas phase to solvents of increasing relative permittivity.

The progressive decrease of the *ax/eq* population ratio as the solvent polarity increases is in line with the experimental IR trend. Moreover, the computed PCM ν_CO_ frequencies of the two conformers do not significantly differ in the non-polar solvents. In contrast, the ν_CO_ frequency of the *eq* conformer becomes progressively smaller than that of the *ax* one as the solvent polarity increases. These results further support the experimental IR frequency assignments. The lower ν_CO_ IR frequency of the *eq* conformer in solution may be rationalised by the analysis of the computed geometries, which indicate that in the *eq* conformer the carbonyl oxygen atom is free for solvation, while in the *ax* conformer the presence of the [O_CO_^…^H*_o_*_-Ph_] hydrogen bond partially prevents the solvent molecules' approach to the carbonyl oxygen atom. Thus, the larger solvation at the carbonyl oxygen atom of the most polar *eq* conformer decreases the carbonyl bond order and consequently its frequency to a larger extent than in the *ax* conformer. 

The sum of the energy contributions of the orbital interactions (NBO analysis) and the electrostatic interactions correlates well with the populations and the ν_CO_ frequencies of both conformers in the gas phase. The smaller contribution of the LP_N_→π*_CO_ orbital interaction for the *eq* conformer with respect to the *ax* conformer is in line with the smaller contribution of the [^−^O‒C=N^+^] canonical form to the resonance hybrid due to the strong Repulsive Field Effect between the C^δ+^=O^δ−^ and C^δ+^-S^δ−^ dipoles, which destabilises the *eq* conformer and increases its ν_CO_ frequency relative to that of the *ax* conformer.

X-ray single crystal analysis of **2** indicates that this compound assumes, in the solid, the most polar *eq* geometry with respect to the [O=C-C-SPh] moiety and that the 2-piperidone ring displays a slightly distorted half-chair conformation. In order to obtain the largest energy gain from the crystal packing, the molecules of the *eq* conformer of **2** are arranged in the solid through a helical fashion due to hydrogen bond interactions, *i.e.*, C(3)-H(5)^…^O(1) and C(7)-H(14)^…^π_(Ph)_.

## Supplementary Materials

Supplementary materials can be accessed at: http://www.mdpi.com/1420-3049/18/7/7492/s1.
