# Supplementary material for: Spectroscopic and Theoretical Studies of Some 3-(4′-Substituted phenylsulfanyl)-1-methyl-2-piperidones
Source: Molecules. 2013 Jun 27;18(7):7492–509. doi: 10.3390/molecules18077492 (PMC6269881; doi:10.3390/molecules18077492)
Supplement: Supplementary file 1 [file molecules-18-07492-s001.pdf]

# Supplementary Materials

**Table S1.** Relative energies (E, kJ mol$^{-1}$), dipole moments (μ, D), carbonyl frequencies (ν, cm$^{-1}$), and selected torsion angles (deg) calculated for the minimum energy conformations of 3-phenylthio-1-methyl-2-piperidone **4** at the MP2/6-31+G(d,p) level.

| Method | Conf.[a] | E[b] | P[c] | μ | $\nu_{CO}$ | Torsion angles/° [d] | | | | | | | |
|---|---|---|---|---|---|---|---|---|---|---|---|---|---|
| | | | | | | α | β | γ | δ | θ | θ' | ω | ω' |
| MP2/6-31+G(d,p) | *ax* | 0 | 77.7 | 3.68 | 1717.5 | 85.2 | −104.3 | 55.5 | 4.16 | 161.8 | −151.1 | 30.3 | −52.6 |
| | *eq* | 3.09 | 22.3 | 5.89 | 1715.7 | 27.1 | 178.9 | 73.0 | −6.20 | −163.1 | 152.8 | −31.1 | 52.2 |

[a] Conformer designations *ax* and *eq* refers to the *axial* and *equatorial* conformers, respectively; [b] Relative energy; [c] Molar fraction in percentage; [d] See Scheme 1.

**Table S2.** Single point PCM solvent effect on the minimum energy gas phase for the conformations of **4** at the MP2/6-31+G(d,p) level; relative energies (E, kJ mol$^{-1}$) and dipole moments (μ, D).

| Conf.[a] | $C_7H_{14}$ | | | $CCl_4$ | | | $CHCl_3$ | | | $CH_2Cl_2$ | | | $CH_3CN$ | | |
|---|---|---|---|---|---|---|---|---|---|---|---|---|---|---|---|
| | E[b] | P[c] | μ | E | P | μ | E | P | μ | E | P | μ | E | P | μ |
| *ax* | 0 | 58.6 | 4.10 | 0 | 53.7 | 4.19 | 1.63 | 34.1 | 4.55 | 2.76 | 24.7 | 4.75 | 3.89 | 17.2 | 4.95 |
| *eq* | 0.86 | 41.4 | 6.59 | 0.37 | 46.3 | 6.74 | 0 | 65.9 | 7.35 | 0 | 75.3 | 7.69 | 0 | 82.8 | 8.04 |

[a] Conformer designations *ax* and *eq* refers to the *axial* and *equatorial* conformers, respectively; [b] Relative energy; [c] Molar fraction in percentage.

**Table S3.** Mülliken charge (*e*) at selected atoms obtained at the B3LYP/6-31+G(d,p) level for 3-phenylsulfanyl-1-methyl-2-piperidone **4**.

| Conf. | O(1) | C(2) | S(4) | N(6) | H(26) | H(30) | H(5) | H(11) | H(16) | H(17) |
|---|---|---|---|---|---|---|---|---|---|---|
| ***ax*** | -0.467 | 0.012 | 0.045 | -0.057 | 0.181 | 0.131 | 0.213 | 0.195 | 0.148 | 0.167 |
| ***eq*** | -0.458 | -0.037 | 0.059 | -0.182 | 0.131 | 0.135 | 0.196 | 0.195 | 0.149 | 0.154 |

**Table S4.** NBO occupancy of selected orbitals calculated at the B3LYP/6-31+G(d,p) level for the *ax* and *eq* conformers of 3-phenylthio-1-methyl-2-piperidone **3**.

| **Orbitals** | ***ax*** | | ***eq*** | |
|---|---|---|---|---|
| | **DO** [a] | **AO** [b] | **DO** | **AO** |
| $LP_{N6}\rightarrow\pi^*_{C2=O1}$ | 1.666 | 0.320 | 1.674 | 0.284 |
| $LP_{O1}\rightarrow\sigma^*_{C2-N6}$ | 1.866 | 0.079 | 1.861 | 0.080 |
| $LP_{O1}\rightarrow\sigma^*_{C2-C3}$ | 1.866 | 0.070 | 1.861 | 0.068 |
| $LP_{O1}\rightarrow\sigma^*_{C21-H26}$ | 1.866 | 0.134 | 1.861 | 0.014 |
| $LP_{O1}\rightarrow\sigma^*_{C10-H11}$ | 1.866 | 0.009 | 1.861 | 0.011 |
| $LP_{S4}\rightarrow\pi^*_{C2=O1}$ | 1.892 | 0.320 | 1.925 | 0.284 |
| $LP_{S4}\rightarrow\pi^*_{C20-C21}$ | 1.892 | 0.388 | 1.925 | 0.368 |
| $\sigma_{C3-S4}\rightarrow\pi^*_{C2=O1}$ | 1.959 | 0.320 | 1.964 | 0.284 |
| $\sigma_{C3-S4}\rightarrow\sigma^*_{C2-N6}$ | 1.959 | 0.079 | 1.964 | 0.080 |
| $\sigma_{C3-S4}\rightarrow\sigma^*_{C9-H18}$ | 1.959 | 0.016 | 1.964 | 0.016 |
| $\pi_{C2=O1}\rightarrow\sigma^*_{C3-S4}$ | 1.989 | 0.037 | 1.989 | 0.034 |
| $\pi^*_{C2=O1}\rightarrow\sigma^*_{C3-S4}$ | 0.320 | 0.037 | 0.284 | 0.034 |

[a,b] Donor and acceptor orbitals, respectively.

ax.gjf

```
%mem=8000MB
%nprocshared=7
# freq rb3lyp/6-31+g(d,p) scrf=check guess=tcheck genchk geom=allcheck

Title Card Required

0 1
 C     1.56098700    0.85633300    0.24008800
 C     3.60080300   -0.59046300   -0.05870300
 C     1.63206400   -1.53087700    1.16724900
 C     2.72495400   -1.82464300    0.13978100
 C     0.75740000   -0.37324400    0.67499300
 N     2.83652200    0.65386700   -0.21726500
 H     4.23122400   -0.70952900   -0.94786400
 H     1.01260000   -2.41162500    1.36015000
 H     3.35978700   -2.65676400    0.46287400
 H     0.07465600   -0.03551400    1.45626300
 H     4.28189100   -0.47554100    0.80057600
 H     2.09274800   -1.23942300    2.12252500
 H     2.26832300   -2.12112500   -0.81228800
 O     1.04343500    1.97548000    0.27788000
 S    -0.32957100   -0.93395300   -0.73712100
 C     3.62304000    1.81149800   -0.63061600
 H     4.13679900    1.58870200   -1.57204700
 H     4.37677800    2.06415700    0.12775900
 H     2.95697900    2.66206400   -0.76435200
 C    -1.94516800   -0.29200300   -0.29125800
 C    -4.53480000    0.63169500    0.25421500
 C    -3.04530900   -1.15043700   -0.43509500
 C    -2.14258700    1.03488500    0.12047000
 C    -3.43530800    1.48214200    0.40537300
```

| | | | |
|---|---|---|---|
| C | -4.33594400 | -0.68352900 | -0.17349400 |
| H | -2.88869400 | -2.18037100 | -0.74155600 |
| H | -1.29298100 | 1.70518100 | 0.21154500 |
| H | -3.58154200 | 2.50811000 | 0.73172200 |
| H | -5.18208700 | -1.35506500 | -0.28933500 |
| H | -5.53727900 | 0.99094700 | 0.46776400 |

eq.gjf

%mem=15500MB
%nprocshared=8
# freq rb3lyp/6-31+g(d,p) scrf=check guess=tcheck genchk geom=allcheck

Title Card Required

0 1

| | | | |
|---|---|---|---|
| C | -2.13586900 | -0.72985600 | 0.16724000 |
| C | -3.08388900 | 1.59452200 | -0.09485700 |
| C | -0.60957500 | 1.28662100 | -0.29250800 |
| C | -1.72165300 | 2.15297600 | 0.30055600 |
| C | -0.72501900 | -0.12752600 | 0.27964600 |
| N | -3.19240100 | 0.14726500 | 0.11790400 |
| H | -3.28495200 | 1.81569500 | -1.15583100 |
| H | -0.70114600 | 1.24919200 | -1.38577200 |
| H | -1.63092900 | 2.17992000 | 1.39423200 |
| H | -0.49769700 | -0.10026100 | 1.35352300 |
| H | -3.87951500 | 2.07739600 | 0.48550500 |
| H | 0.37634700 | 1.70258000 | -0.06662500 |
| H | -1.64555100 | 3.18705900 | -0.05359200 |
| O | -2.29786500 | -1.94857800 | 0.18249200 |
| S | 0.44377000 | -1.35141500 | -0.48961600 |
| C | -4.54168000 | -0.39595000 | -0.00451000 |
| H | -4.93140000 | -0.24552800 | -1.02055500 |
| H | -5.20856400 | 0.10936500 | 0.70282600 |
| H | -4.51473400 | -1.46275300 | 0.21018800 |
| C | 2.01294800 | -0.53285300 | -0.16994000 |
| C | 4.52594500 | 0.63480000 | 0.29342200 |
| C | 2.74878100 | 0.01246700 | -1.23191700 |
| C | 2.55348000 | -0.50050800 | 1.12551400 |

| | | | |
|---|---|---|---|
| C | 3.79701600 | 0.09137500 | 1.35648100 |
| C | 4.00255400 | 0.58722700 | -1.00080300 |
| H | 2.33666700 | -0.01824500 | -2.23576500 |
| H | 2.00465500 | -0.95252400 | 1.94664100 |
| H | 4.20420800 | 0.11266000 | 2.36350800 |
| H | 4.56563600 | 1.00142300 | -1.83247600 |
| H | 5.49814200 | 1.08465100 | 0.47284300 |
